# Cytoplasmic mislocalization of RNA splicing factors and aberrant neuronal gene splicing in TDP-43 transgenic pig brain

**DOI:** 10.1186/s13024-015-0036-5

**Published:** 2015-09-03

**Authors:** Guohao Wang, Huaqiang Yang, Sen Yan, Chuan-En Wang, Xudong Liu, Bentian Zhao, Zhen Ouyang, Peng Yin, Zhaoming Liu, Yu Zhao, Tao Liu, Nana Fan, Lin Guo, Shihua Li, Xiao-Jiang Li, Liangxue Lai

**Affiliations:** State Key Laboratory of Molecular Developmental Biology, Institute of Genetics and Developmental Biology, Chinese Academy of Sciences, Beijing, 100101 China; Key Laboratory of Regenerative Biology, South China Institute for Stem Cell Biology and Regenerative Medicine, Guangzhou Institutes of Biomedicine and Health, Chinese Academy of Sciences, Guangzhou, 510530 China; Department of Human Genetics, Emory University School of Medicine, Atlanta, GA 30322 USA; University of Chinese Academy of Sciences, Beijing, 100049 China

**Keywords:** TDP-43, PSF, NeuN, NMHC II-B, ALS, transgenic Pig

## Abstract

**Background:**

TAR DNA-binding protein 43 (TDP-43) is a nuclear protein, but it is redistributed in the neuronal cytoplasm in both amyotrophic lateral sclerosis (ALS) and frontotemporal lobar degeneration (FTLD). Because small transgenic animal models often lack cytoplasmic TDP-43, how the cytoplasmic accumulation of TDP-43 contributes to these diseases remains unclear. The current study is aimed at studying the mechanism of cytoplasmic pathology of TDP-43.

**Results:**

We established transgenic pigs expressing mutant TDP-43 (M337V). This pig model shows severe phenotypes and early death. We found that transgenic TDP-43 is also distributed in the cytoplasm of neuronal cells in the spinal cord and brain. Transgenic TDP-43 interacts with PSF, an RNA splicing factor that associates with NeuN to regulate neuronal RNA splicing. The interaction of TDP-43, PSF and NeuN causes PSF and NeuN mislocalize into the neuronal cytoplasm in transgenic pigs. Consistently, abnormal PSF-related neuronal RNA splicing is seen in TDP-43 transgenic pigs. The cytoplasmic localization of PSF and NeuN as well as abnormal PSF-related neuronal RNA splicing was also found in ALS patient brains.

**Conclusion:**

Our findings from a large mammalian model suggest that cytoplasmic mutant TDP-43 could reduce the nuclear function of RNA splicing factors, contributing to neuropathology.

## Background

TAR DNA-binding protein 43 (TDP-43) is a multifunctional, nuclear protein that binds RNA to regulate RNA processing, including alternative splicing, miRNA production, and mRNA trafficking and stabilization [1–6]. TDP-43 has two conserved RNA recognition motifs and a C-terminal glycine-rich domain that mediates interactions with other heterogeneous nuclear ribonucleoprotein (hnRNP) family members [7, 8]. Mutations in the C-terminal domain of TDP-43 are associated with both amyotrophic lateral sclerosis (ALS) and frontotemporal lobar degeneration (FTLD) [9–11]. ALS is characterized by progressive degeneration of large motor neurons in the spinal cord and cerebral cortex, and FTLD causes degeneration of neurons in the frontal and temporal cortices. In both diseases, the key pathological change is that pathogenic TDP-43 is accumulated in the cytoplasm and forms cytoplasmic aggregates [12–14]; however, how cytoplasmic mislocalization of TDP-43 contributes to pathogenesis remains to be investigated.

To date, a variety of transgenic TDP-43 animal models have been established and provided us with valuable tools to uncover gene expression alteration and aberrant RNA splicing caused by TDP-43 [5, 7, 15]. Although overexpression of mutant TDP-43 in cultured cells and some rodent models and deletion of the nuclear localization signal in TDP-43 can lead to the cytoplasmic distribution of mutant TDP-43, many transgenic TDP-43 mouse models fail to show the cytoplasmic accumulation of mutant TDP-43 [16–24], suggesting that species-dependent differences may account for differential pathology of TDP-43 in small animals and human patients. Given that TDP-43 is a multifunctional nuclear protein whose expression is tightly regulated [25], mutant TDP-43 or altered levels of normal TDP-43 can cause neuropathology. However, because most of current animal models lack the cytoplasmic accumulation of TDP-43, our ability to investigate how the cytoplasmic mislocalization of TDP-43 contributes to the pathogenesis has been limited.

The lack of cytoplasmic mislocalization of TDP-43 in small animals may be due to unknown species differences that regulate the subcellular localization of TDP-43. Because of the greater similarity between pigs and humans, we have used transgenic pigs to investigate the pathology caused by mutant polyglutamine proteins and SOD1 and uncovered unique pathology not seen in small animals [26, 27]. In the current study, we established transgenic miniature pigs expressing mutant TDP-43 and found that transgenic TDP-43 is also distributed in the cytoplasm of neuronal cells and causes severe phenotypes in pigs. Moreover, this new animal model demonstrates that mutant TDP-43 interacts with the RNA splicing factor PSF (Splicing Factor Proline/Glutamine-Rich) and the cytoplasmic localization of PSF as well as reducing nuclear PSF function in RNA splicing. Thus, by establishing a large mammalian model of TDP-43, we provide fresh insight into the role of cytoplasmic mislocalization of mutant TDP-43 in the pathogenesis of ALS and FTLD.

## Results

### Generation of transgenic pigs expressing mutant TDP-43

**Fig. 1:**
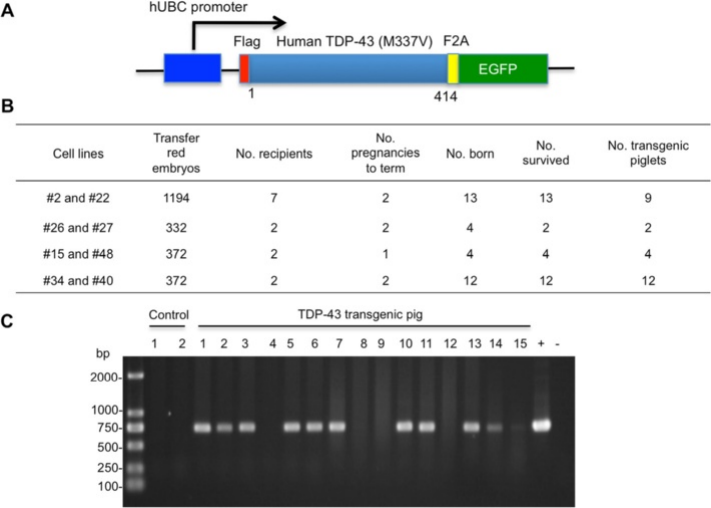
Generation of transgenic TDP-43 pigs. **a** The plasmid DNA structure for expressing mutant TDP-43 (M337V) under the human ubiquitin promoter in transgenic pigs. F2A is a peptide sequence that can self-cleave to separate TDP-43 from EGFP in cells. **b** The numbers of embryos used and transgenic TDP-43 pigs produced. **c** PCR genotype analysis of ear tissues from 15 transgenic TDP-43 pigs. Two non-transgenic pig (Control) ear tissues served as a control

There are a number of mutations in human TDP-43, and transgenic expression of wild type human TDP-43 also causes neuropathology in animal models [22, 28]. Considering that generation of transgenic pigs is much more time-consuming and costly than small animal models, we focused on the generation of transgenic pigs expressing human mutant TDP-43 (M337V), but not wild type TDP-43, and compared them with non-transgenic pigs. Using the same somatic cell nuclear transfer (SCNT) approach for generating transgenic pig models of Huntington’s disease and ALS [26, 27], we transfected mutant TDP-43 (M337V) in primary pig fetal fibroblasts isolated from 35-day-old Tibetan miniature pig fetus. Mutant TDP-43 is tagged with the flag epitope at its N-terminus and expressed under the control of the human ubiquitin promoter that can drive gene expression ubiquitously, and the transgene also co-expresses EGFP for identifying transfected cells that express TDP-43 (Fig. 1a). The transfected cells expressing TDP-43 were fused to a total of 2270 SCNT embryos, which were transferred into 13 surrogates that exhibited natural estrus. Seven surrogates developed to term and gave birth to 31 healthy Tibetan miniature piglets after 120–130 d of gestation. Of these, 27 piglets were found to be positive for transgenic TDP-43 via PCR (Fig. 1b), as PCR analysis of DNA samples obtained from the ear tissues of piglets showed that the live piglets were positive for the TDP-43 transgene (Fig. 1c).

**Fig. 2:**
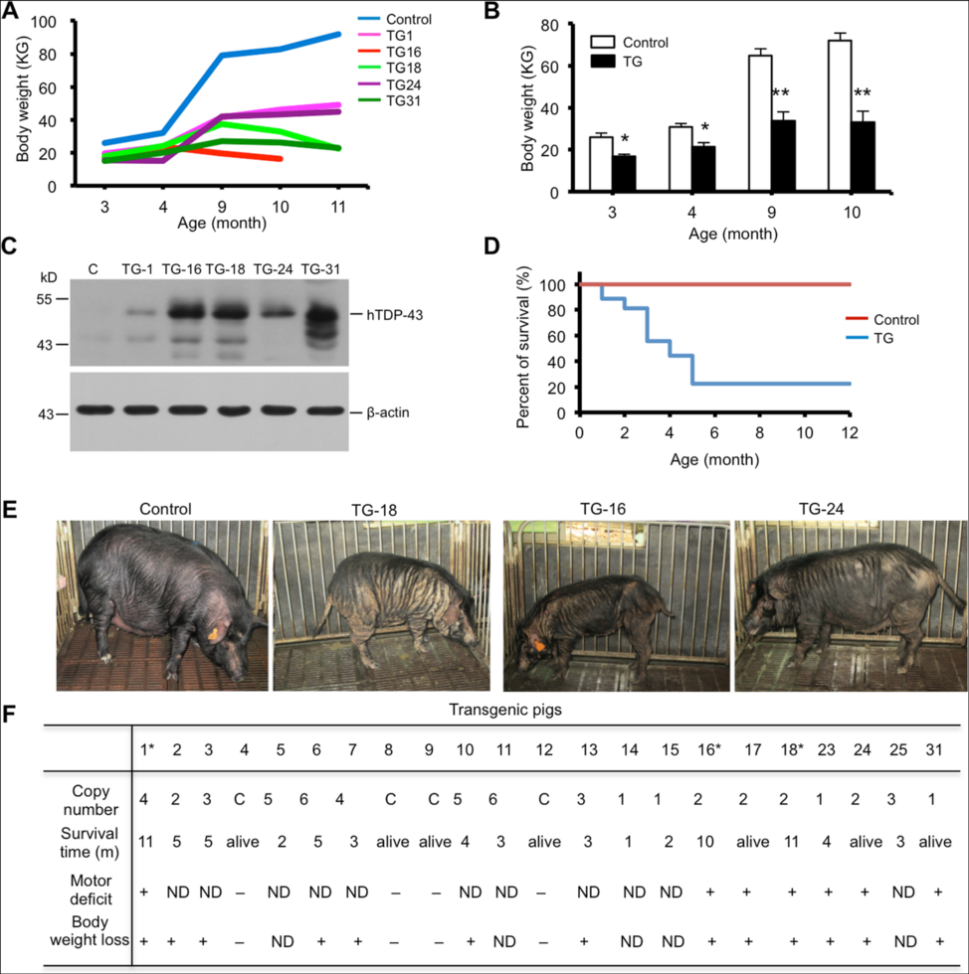
Progressive phenotypes of transgenic TDP-43 pigs. **a** Body weight reduction in different transgenic TDP-43 pigs at different ages. **b** Body weight (mean ± SE) of non-transgenic and transgenic TDP-43 pigs at 3, 4, 9 and 10 months of age (n = 5 for non-TG (Control) and n = 5 for TDP-43 transgenic pigs (TG)). * *p* < 0.05; ** *p* < 0.01. **c** Western blots analysis of ear tissues showing the expression of transgenic TDP-43 pigs that were monitored for their body weights. **d** Survival plot showing that expression of mutant TDP-43 caused early death of TDP-43 transgenic (TG) pigs (n = 12 for non-TG (Control) and n = 27 for TG). **e** Photographs of representative non-transgenic (Control) and TDP-43 transgenic pig (TG-16, 18, 24) at 9 months of age. **f** Summary of some transgenic pigs for their genotypes and phenotypes. C: Genotype is non-transgenic with the endogenous copies of pig TDP-43

Newborn transgenic TDP-43 pigs appeared as normal at birth as non-transgenic pigs. However, they started to gain less body weight from 3 months after birth compared to non-transgenic pigs (Fig. 2a and b). The extent of body weight loss appears to be correlated with the expression levels of TDP-43 in pig ear tissues (Fig. 2c). Many transgenic TDP-43 pigs die earlier starting at the age of 4–5 months, and 85 % (21/27) of the TDP-43 transgenic pigs died within one year (Fig. 2d). As a result of gaining considerable less body weight during development, the transgenic TDP-43 pigs often displayed loose skin characterized by wrinkled and sagging skin on their bodies (Fig. 2e). We previously established ALS pigs expressing mutant SOD1, which grew normally without body weight loss but showed an obvious running defect on a treadmill [27]. Like these ALS transgenic pigs, the TDP-43 transgenic pigs also showed progressive weakness and limb movement defects (Fig. 2f). However, following the training for the treadmill test, some TDP-43 pigs died, which did not allow us to use the treadmill to obtain quantified data of the limb movement impairment for TDP-43 pigs. Therefore, we used the surviving TDP-43 pigs, not including those that died right after treadmill test, to assess the growth, life span, and motor deficit of TDP-43 pigs. We found that there was the progressive reduction in the body weight and that transgenic TDP-43 pigs died earlier than non-transgenic pigs. The shorter life span and the more severe motor deficit in TDP-43 pigs than those of our previously reported transgenic SOD1 pigs [27] suggest that mutant TDP-43 elicits more toxicity than mutant SOD1 in transgenic pigs.

**Fig. 3:**
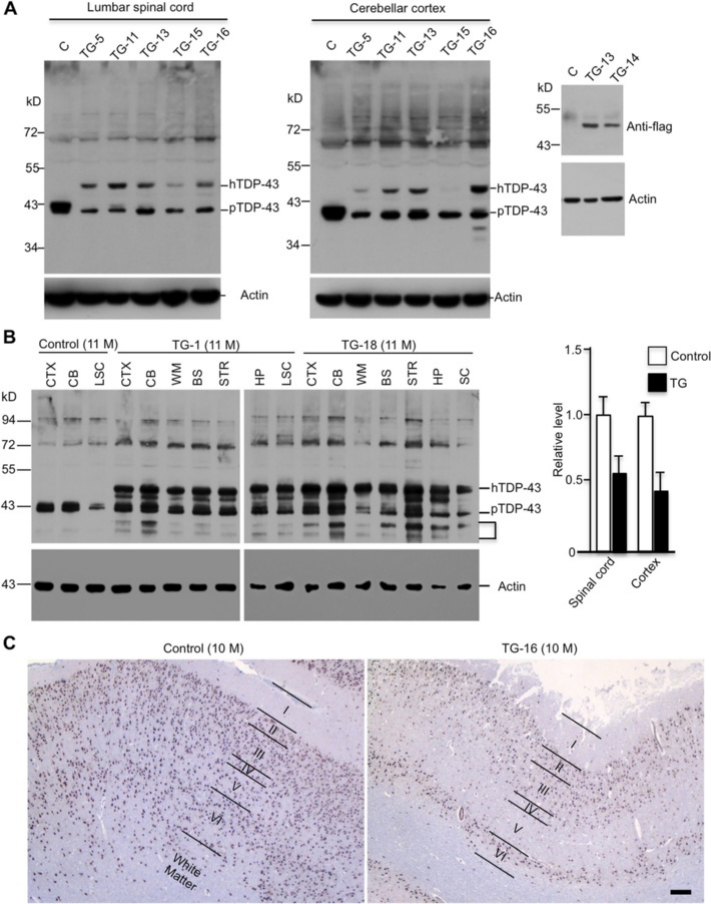
Expression of transgenic TDP-43 in the pig brain tissues. **a** Western blot analysis the brain cortex and spinal cord tissues of several transgenic TDP-43 pigs using antibodies to TDP-43 and flag. C: non-transgenic. **b** Comparison of the transgenic TDP-43 expression in different brain and peripheral tissues in TG-11 and TG-18 pigs. Bracket indicates degraded fragments of transgenic TDP-43. CTX, cortex; CB, cerebellum; LSC, lumbar spinal cord; WM, white matter; BS, brain stem; STR, striatum; HP, hippocampus; SC, spinal cord. Right panel shows quantification of the ratios of endogenous pig TDP-43 to actin in non-transgenic (Control) pig brains and transgenic TDP-43 to actin on western blots (mean ± SE, n = 5 pigs per group). **c** The brain cortex from non-transgenic (Control) and TDP-43 transgenic pig (TG-16) pigs was stained with anti-NeuN. Layers of the pig brain cortex were indicated, showing a reduced thickness of transgenic TDP-43 pig brain cortex compared to the non-transgenic pig brain cortex. Scale bar: 200 μm

Of the dead TDP-43 pigs, we were able to collect brain tissues from some animals for western blotting and immunohistochemical studies. Using anti-TDP-43 (G400, Cell Signaling), which recognizes endogenous pig TDP-43 and flag-tagged transgenic TDP-43 via western blotting, we found that the levels of transgenic TDP-43 in the spinal cord and cerebellar cortex tissues in different transgenic pigs are lower than or equivalent to the endogenous pig TDP-43 (Fig. 3a). Also, transgenic TDP-43 is widely expressed in different brain regions, including the cerebellum, striatum, and brain stem (Fig. 3b). It is known that the expression level of TDP-43 is tightly self-regulated [29]. By measuring the ratios of endogenous pig TDP-43 to actin in non-transgenic pigs and mutant TDP-43 to actin in transgenic TDP-43 pig brains, we found that the expression levels of mutant TDP-43 are not overexpressed (Fig. 3b). Western blotting also revealed small TDP-43 immunoreactive bands (Fig. 3b), which are likely cleaved transgenic TDP-43 fragments as they were not seen in the non-TG control pig brain. In the pig brain cortex, we saw a reduction in the thickness of different layers [(the thickness of layers II-VI (non-TG 1396.9 ± 51.67 μm vs TG 1038.05 ± 36.49 μm (mean ± SE, n = 4)] with a reduced density of NeuN-positive cells in the TDP-43 transgenic pig brain (Fig. 3c), which indicates neurodegeneration in TDP-43 transgenic pig brains.

**Fig. 4:**
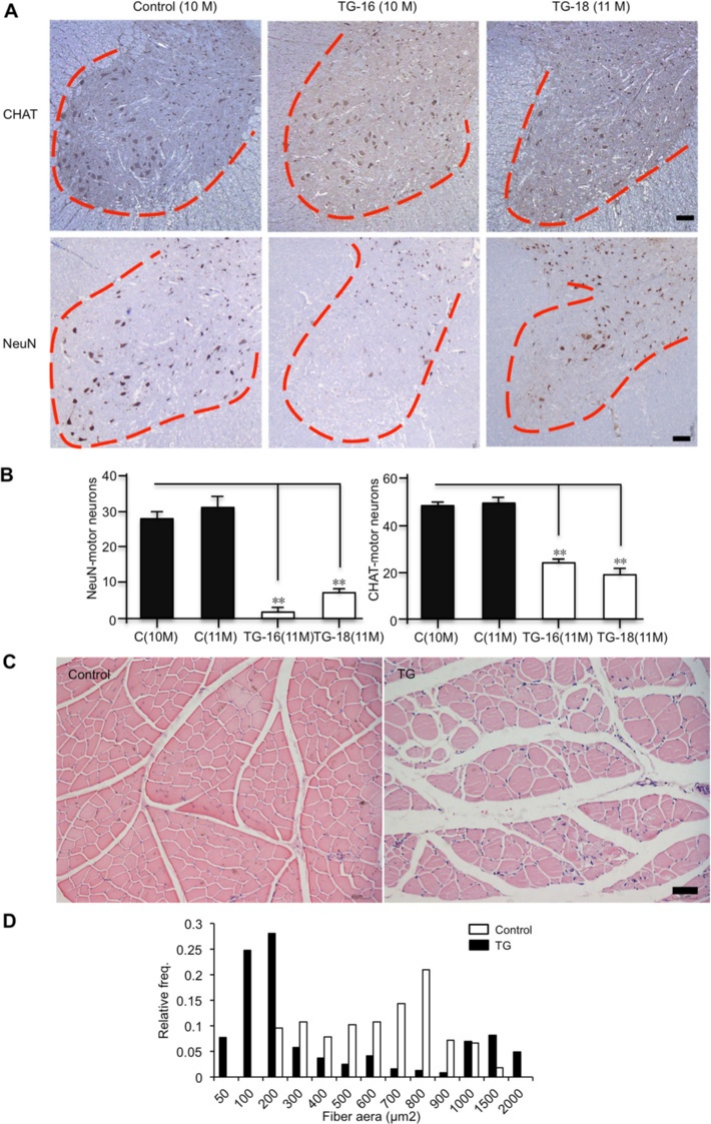
Expression of mutant TDP-43 in transgenic pigs causes neurodegeneration and muscle atrophy. **a** CHAT- and NeuN-immunostaining of the pig spinal cord showing a reduced number of motor neurons in TDP-43 transgenic pigs (TG-16 and 18) at 10–11 months of age. Control: non-transgenic. Scale bars: 200 μm. **b** Quantitative analysis of the number (mean ± SE) of NeuN- and CHAT-positive neurons per ventral horn in the spinal cord sections. Five-eight images obtained with 20x N.A. 0.8 objective per section and 5 sections per group were analyzed. C: non-transgenic. **c** Skeletal muscle atrophy in TDP-43(M337V) transgenic pigs. Haematoxylin and eosin-stained cross-sections gastrocnemius muscles from 10-month-old non-transgenic (Control) and TDP-43(M337V) transgenic (TG-16) pigs. Scale bar: 50 μm. **d** More than 3 random fields (corresponding to 200–300 cross-sectioned fibers) were examined for quantification of muscle fibers of different sizes

We know that mutant TDP-43 affects the spinal cord motor neurons to cause ALS [30, 31], so we examined the numbers of motor neurons in the ventral horn of lumbar spinal cords via immunostaining with antibodies to NeuN and CHAT, which is specifically expressed in motor neurons. There were fewer NeuN-positive and CHAT-positive motor neurons (Fig. 4a) in the spinal cord of TDP-43 transgenic pigs. Counting the numbers of these neurons showed that motor neurons were decreased obviously in the ventral horn of lumbar spinal cord of transgenic pigs (Fig. 4b). The degeneration of spinal cord motor neurons causes denervated skeletal muscle in ALS patients. Similarly, we found that size of muscle fibers is markedly reduced in transgenic TDP-43 pigs (Fig. 4c, d). Examination of other peripheral tissues such as heart, liver, stomach, intestines, and kidney did not reveal any significant difference between non-transgenic and transgenic TDP-43 pigs. Although we cannot rule out that the peripheral toxicity of transgenic TDP-43 also contributes to the severe phenotypes of transgenic pigs, the evidence for neurodegeneration in transgenic TDP-43 pigs indicates the important role of neuronal toxicity in transgenic TDP-43 and led us to further explore the mechanism underlying the neurodegeneration.

### Interaction of TDP-43 with PSF in the cytoplasm

**Fig. 5:**
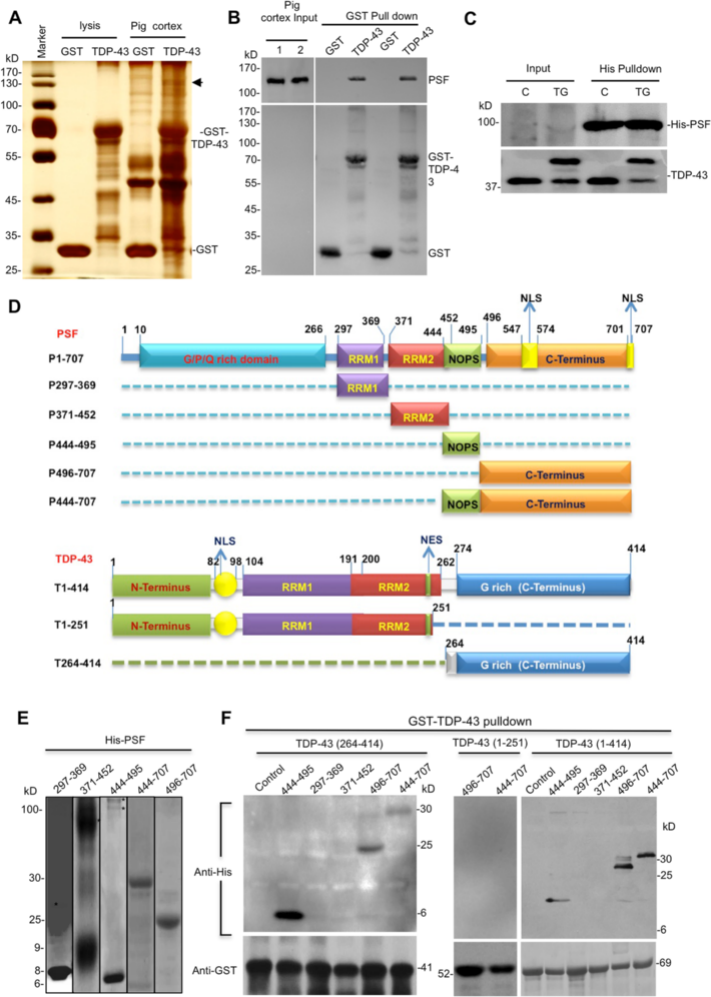
TDP-43 interacts with PSF and NeuN. **a** Silver-staining of 4-12 % SDS-PAGE gel showing GST-TDP-43 pull-down from pig cortex lysates. GST served as a control. The indicated band was analyzed by mass spectrometry and found to contain PSF. **b** Western blotting with anti-PSF verifying the interaction of GST-TDP-43 and PSF in pig brain cortex lysates. Two GST and GST-TDP-43 pull-down proteins were analyzed. **c** Purified His-PSF was incubated with transgenic pig brain cortex lysates, and the bound proteins were subjected to western blot, which shows the in vitro direct interaction of endogenous pig TDP-43 and transgenic human TDP-43 with His-PSF. Input is 5 % of purified His-PSF, and 40 % of pulldown was loaded into the SDS gel. **d** PSF contains N-terminal, RRM1, RRM2, and C-terminal domains. The N-terminal domain is a glycine/proline/arginine rich domain, the C-terminal and the NOPS regions of PSF were found to bind to TDP-43. TDP-43 contains N-terminal, RRM1, RRM2, and C-terminal (G-rich) domains. **e** Purification of His-PSF containing different domains as indicated with the corresponding amino acid positions. **f** In vitro GST-pull down identifying that the C-terminal regions containing amino acids 444–707 of PSF bind to C-terminal GST-TDP-43 (amino acids 264–414), but not N-terminal TDP-43 (amino acids 1–251)

TDP-43 is involved in gene transcription and RNA splicing via its interactions with other proteins. To identify any pig proteins that could interact with TDP-43 and are involved in TDP-43-mediated toxicity in transgenic pigs, we generated GST-TDP-43 and used it to pull down pig brain cortex proteins. The GST pull-down uncovered a band that is specific to GST-TDP-43 pull-down (Fig. 5a). Via mass spectrometry, this band was identified as the splicing factor proline-glutamine rich (SFPQ/PSF) or PSF, an essential pre-mRNA splicing factor required in spliceosome formation [32–34]. Western blot analysis confirmed the interaction of PSF in pig brain with GST-TDP-43 (Fig. 5b). We also generated His-tagged PSF and purified this recombined protein from bacteria. In vitro binding showed that His-PSF could bind endogenous pig TDP-43 and transgenic hTDP-43 in the pig brain cortex lysates (Fig. 5c). Like TDP-43, PSF contains N-terminal, RRM1, RRM2 and C-terminal domains. To define the binding region in TDP-43, we generated GST fusion proteins containing different TDP-43 fragments (1–251, 1–414, and 264–414 amino acids) and used these GST fusion proteins to incubate with His-PSF proteins containing different regions (Fig. 5d). The results revealed that C-terminal PSF (444–707 amino acids) binds GST fusion protein containing C-terminal (264–414 amino acids) TDP-43 (Fig. 5e and f).

**Fig. 6:**
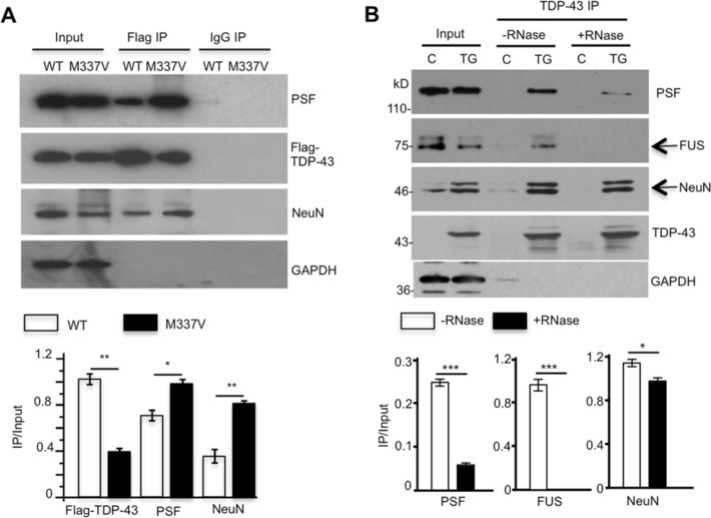
Interactions of TDP-43 with PSF and NeuN. **a** Representative western blots showing co-immunoprecipitation of transfected flag-TDP-43 (WT or mutant TDP-43 (M337V)) by anti-flag and endogenous PSF, NeuN in mouse N2A cells. IgG immunoprecipitation served as controls. The ratios of precipitated to input were obtained from 3 independent experiments and shown beneath the blots. **b** The *in vivo* interaction of TDP-43 (M337V) with PSF and NeuN in the non-transgenic (C) pig and transgenic TDP-43 (TG) pig brain. The immunoprecipitation was also pre-treated with RNase to remove RNA. An antibody to TDP-43 (12892-1-AP from Proteintech) selectively detected human TDP-43, but not endogenous pig TDP-43. The interaction of FUS with TDP-43, which depends on RNA, served as a control. Quantitative data (ratio of IP/input) are presented beneath the blots. IP bands indicated by arrows were used for quantification. *p < 0.05, **p < 0.01 and ***p < 0.001 compared with –RNase treatment

PSF is found to interact with NeuN to activate neural cell-specific alternative splicing [35]. We then examined the effect of transfected TDP-43 on the association of PSF with NeuN. Immunoprecipitation of transfected TDP-43 in mouse N2A cells revealed that mutant TDP-43 (M337V) bound more PSF and NeuN than did wild-type TDP-43 (Fig. 6a). The in vivo interaction of TDP-43 with PSF/NeuN in the pig brain lysates was also verified via immunoprecitation with an antibody to TDP-43 (12892-1-AP from Proteintech) that selectively precipitated and detected human TDP-43 but not endogenous pig TDP-43 (Fig. 6b). We also treated the lysates with RNase to remove RNA before immunopreciptation. Depletion of RNA by this RNase treatment significantly diminished the interactions of TDP-43 with FUS, which is a RNA-binding protein whose interaction with TDP-43 depends on RNA [6, 36, 37]. There was also a slight decrease in the association of TDP-43 with PSF, but not NeuN, compared to those without RNase treatment (Fig. 6b), suggesting that the interactions of TDP-43 with its partners are differentially influenced by RNA in cells.

### Mutant TDP-43 causes the cytoplasmic mislocalization of PSF and NeuN

**Fig. 7:**
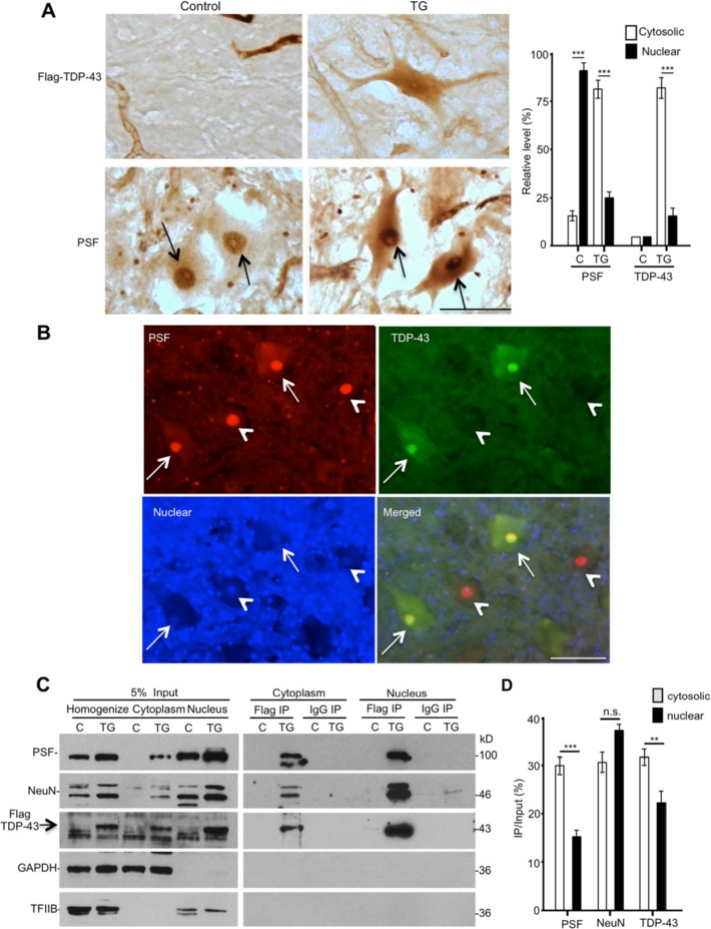
Cytoplasmic mislocalization of PSF and TDP-43 in the spinal cord of transgenic pigs. **a** TDP-43 and PSF antibody immunocytochemistry of the spinal cord regions of non-transgenic (Control) pig and TDP-43 transgenic (TG-23) pigs. Arrows indicate the nuclei of neurons. Scale bar: 50 μm. **b** Immunofluorescent labeling of the spinal cord sections of the TDP-43 pig (TG-23) showing that in motor neurons (arrows) expressing transgenic TDP-43 (green), cytoplasmic distribution of PSF (red) is also evident. The nuclei were stained with Hoechst. Arrows indicate neurons in which transgenic TDP-43 is localized in both the nucleus and cytoplasm. Note that the nuclei (arrowheads) of large motor neurons were weakly labeled by Hoechst. Scale bar: 50 μm. **c** Flag-TDP-43 immunoprecipitation of the spinal cord sections of the TDP-43 pig revealing that PSF and NeuN were co-precipitated with cytoplasmic TDP-43 from transgenic pig tissues. C: non-transgenic. Rabbit IgG immunoprecipitation and non-transgenic pig brain tissues served as a control. **d** The relative levels (mean ± SE) of proteins (the ratio of IP/Input on the same blot) are presented in the right panel (n.s.: no significant difference; *p < 0.05; **p < 0.01; ***p < 0.001)

After verifying the in vivo association of TDP-43 with PSF and NeuN, we wanted to know whether their subcellular localization is altered in TDP-43 transgenic pig brain. We performed immunocytochemical studies of the spinal cord of transgenic TDP-43 pigs and observed that TDP-43 was concentrated in the nucleus and also distributed in the cytoplasm (Fig. 7a). Immunostaining of PSF in the same tissue showed more cytoplasmic staining of PSF in neuronal cells in the spinal cord of transgenic TDP-43 pigs (Fig. 7a). To validate whether cytoplasmic TDP-43 causes more PSF to mislocalize in the cytoplasm, we performed double immunofluorescent staining. In those neurons that showed cytoplasmic transgenic TDP-43, cytoplasmic transgenic PSF was indeed present (Fig. 7b). Counting neurons in the spinal cord of two non-transgenic (78 neurons) and three TDP-43 pigs (128 neurons) showed that more neurons (32.3 %) in TDP-43 pig spinal cord displayed a strong cytoplasmic distribution of PSF than those in non-transgenic pigs (4.5 %). To provide additional evidence for this cytoplasmic distribution, we performed western blot analysis of TDP-43 immunoprecipitation of cytoplasmic and nuclear fractions of transgenic TDP-43 pig brain cortex tissues. Transgenic mutant TDP-43 was precipitated with anti-flag so its specific precipitation can be verified by comparing with non-transgenic pig tissues. We also saw the distribution of mutant TDP-43, PSF, and NeuN in the cytoplasmic fraction from TDP-43 transgenic pig brain tissue (Fig. 7c and d). Thus, both immunocytochemical and immunoprecipitation results support the idea that transgenic mutant TDP-43 in the cytoplasm caused PSF and NeuN to mislocalize in the cytoplasm via their interactions.

### Mutant TDP-43 affects PSF/NeuN-mediated RNA splicing

**Fig. 8:**
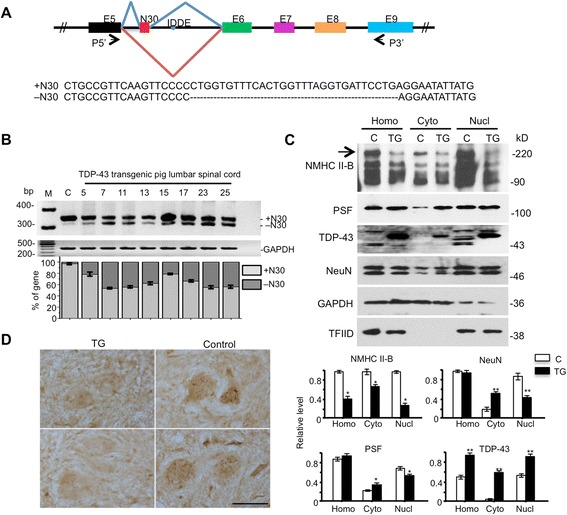
Mutant TDP-43 causes abnormal splicing of NMHC II-B and promotes NMHC II-B degradation. **a** Diagram of the mouse NMHC II-B gene from exon 5 to exon 9. The exons are shown as boxes and the lines between exons are introns. The IDDE sequence is the NeuN binding region, and the alternative splicing exon N30 is indicated as red box between exon 5 and exon 6. The P5’ and P3’ represent the PCR primers to identify N30 inclusion (+N30) or exclusion (−N30). **b** RT-PCR gel result (upper panel) and quantification (lower panel) of the inclusion (+N30) and exclusion (−N30) of exon N30 in the brain cortex of non-transgenic and transgenic TDP-43 pigs (TG-5, −7, −11, −13, −15, −17, −23, −25). GAPDH served as a control. Relative +N30 and -N30 isoform levels are shown below each lane and are normalized by the total NMHC II-B gene amount. **c** Western blot analysis is unable to distinguish +N30 and –N30 because of their few amino acid differences but shows a decrease of NMHC II-B in homogenates, cytoplasmic, and nuclear fractions from transgenic TDP-43 pig cortex compared with non-transgenic (C) pig. GAPDH and TFIID are cytoplasmic and nuclear marker proteins, respectively. The relative levels of NMHC II-B, NeuN, PSF, and TDP-43 in the homogenates (Hom) (ratios of homogenate proteins to GAPDH) as well as cytosolic (Cyto) (ratios of cytoplasmic proteins to GAPDH) and nuclear (ratios of nuclear proteins to TFIID) fractions are presented beneath the blot. The data are mean ± SE (n = 3 animals per group). *p < 0.05; **p < 0.01 compared with non-Tg. **d** Reduced NMHC II-B immunostaining in the motor neurons in the spinal cord of transgenic TDP-43 (TG) pig compared with non-Tg (Control) pig. Two representative images are shown. Scale bar: 50 μm

The interaction of TDP-43 with PSF/NeuN led us to focus on alternative splicing of NMHC II-B (myh 10), nonmuscle myosin heavy chain II-B whose splicing is regulated by PSF and NeuN [35] and is important for neuronal function via regulation of NMDA receptor trafficking [38–40]. NMHC II-B is alternatively spliced via PSF/NeuN to include or exclude a small exon encoding 30 nucleotides, resulting in NMHC II-B +N30 or –N30 isoforms (Fig. 8a). Using RT-PCR analysis, we found that there was more –N30 isoform exclusion in the brain of TDP-43 transgenic pig (Fig. 8b). To examine whether this abnormal splicing affects the expression of NMHC II-B at the protein level, we performed western blotting analysis of cytosolic and nuclear fractions of the pig brain cortex. We were unable to obtain freshly isolated pig brain tissues for fractionation such that the frozen pig brain tissues may affect the separation of subcellular compartments. However, by comparing non-transgenic and transgenic TDP-43 pig brain fractionation samples, we found that transgenic TDP-43 pig brain showed decreases of NMHC II-B in the total, nuclear, and cytoplasmic fractions, indicating that mutant TDP-43 reduces the level of NMHC II-B (Fig. 8c). To confirm that this decrease also occurred in spinal cord, we performed immunostaining of transgenic TDP-43 pig spinal cord and observed that the NMHC II-B staining of motor neurons is much weaker than that in non-transgenic neurons (Fig. 8d).

**Fig. 9:**
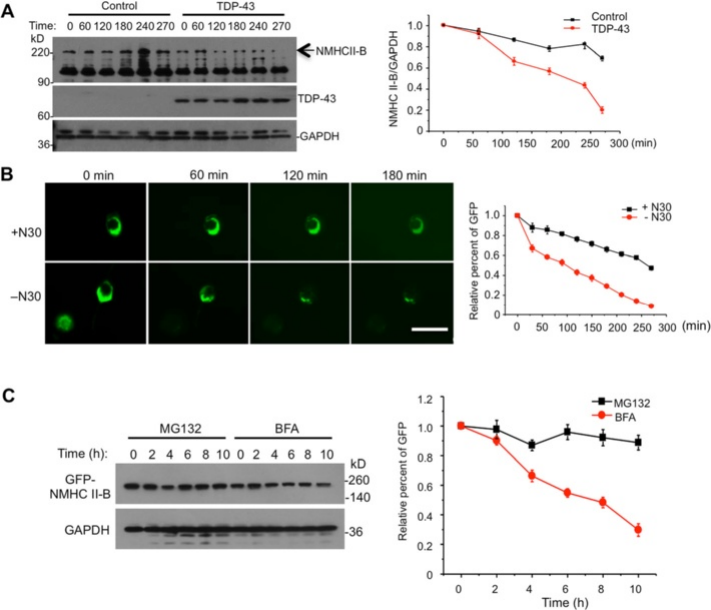
Mutant TDP-43 increases the degradation of NMHC II-B. **a** The half-life of NMHC II-B after cycloheximide (20 ng/ml) treatment of PC12 cells that were transfected with TDP-43 (M337V). Immunoblots were probed with anti-NMHC II-B for detecting endogenous NMHC II-B, anti-Flag for the transfected TDP-43, and anti-GAPDH for a loading control protein. Arrow indicates NMHC II-B and the band above 90 kDa is a non-specific protein reactive to anti-NMHCII-B. Quantification data of % of 0’ time point are presented in the right panel (n = 3). **b** Fluorescent images of transfected N2A cells expressing GFP-NMHC II-B (+N30) or (−N30) after cycloheximide treatment. The right panel shows quantitative fluorescent signals of transfected proteins over time and demonstrates that (−N30) isoform is degraded faster than (+N30). Scale bar: 20 μm. **c** Western blot (the left panel) and quantification analysis (the right panel) of transfected GFP-NMHC II-B (−N30) degradation in the N2A cells that were treated with cycloheximide (20 ng/ml) and MG132 (5 μM) to inhibit the proteasome or BFA (100 μM) to inhibit autophagy

To prove a causal role of TDP-43 in reducing NMHC II-B levels, we transfected mutant TDP-43 in PC12 cells and then examined the half-life of endogenous NMHC II-B using cycloheximide treatment. The transfection of TDP-43 promoted the degradation of endogenous NMHC II-B in PC12 cells (Fig. 9a). Because NMHC II-B consists of +N30 and –N30 isoform and mutant TDP-43 promotes the generation of –N30, we wanted to compare the half-life of +N30 and –N30. Thus, we generated GFP-NMHC II-B (+N30 and-N30) isoforms and expressed them in transfected N2A cells, which allows for high transfection efficiency of this fusion protein. We found that –N30 is degraded faster than +N30 (Fig. 9b). Also, the degradation of –N30 is inhibited by the proteasome inhibitor MG132, but not the autophagy inhibitor BFA (Fig. 9c), suggesting its degradation is mediated by the ubiquitin-proteasome system (UPS). Taken together, our results suggest that TDP-43 affects PSF/NeuN-mediated splicing of NMHC II-B and leads to more –N30 isoform being generated, which is unstable and degraded quickly by the UPS, resulting in a reduced level of NMHC II-B.

### Cytoplasmic mislocalization of TDP-43 and NMHC II-B expression in ALS patient brains

**Fig. 10:**
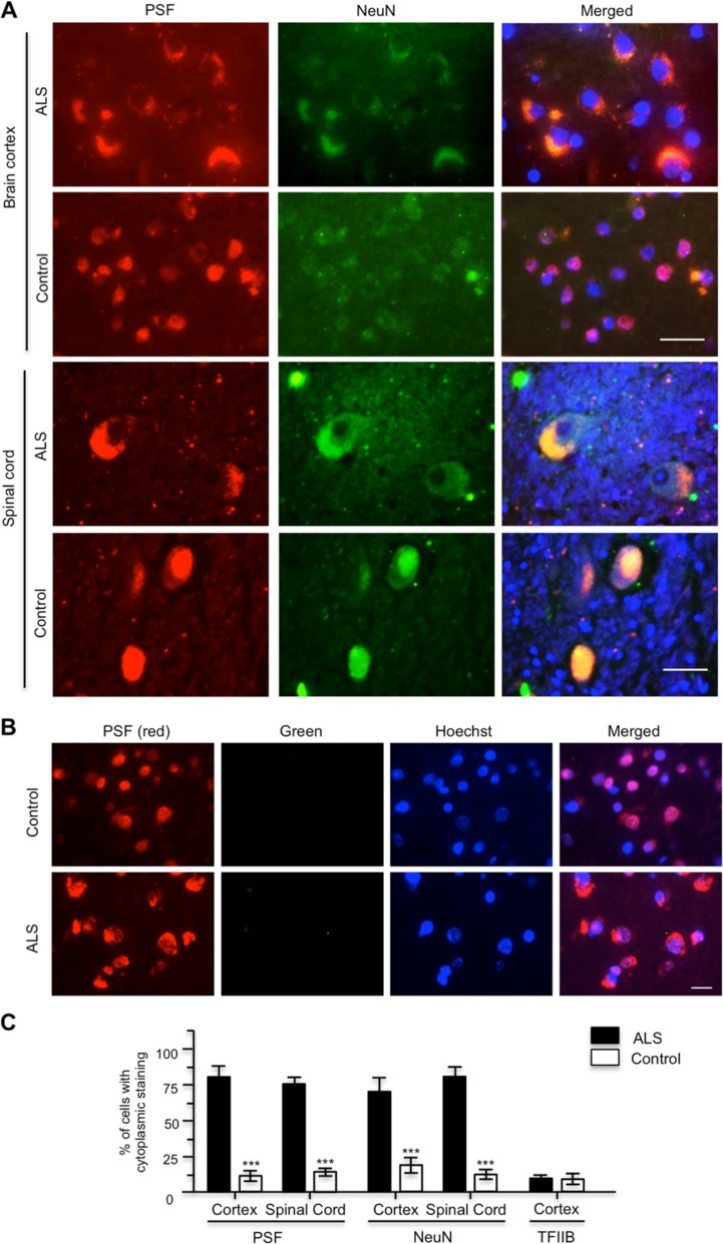
Cytoplasmic mislocalization of PSF, reduction of NMHC II-B, and abnormal splicing of NMHC II-B in the brains of ALS patients. **a** Double immunofluorescent staining of the cortex and spinal cord from the ALS patient and non-ALS individual (control) with antibodies to PSF and NeuN. Scale bars: cortex: 10 μm. Spinal cord: 50 μm. Note that ALS patient brain shows cytoplasmic distribution of PSF and NeuN. **b** Immunostaining of the brain cortex in control and ALS patients with single anti-PSF only revealed PSF immunofluorescent staining. **c** Quantitative analysis of the percentage of cells with cytoplasmic PSF and NeuN staining. The staining of the nuclear protein TFIIB served as controls. The data are mean ± SE by counting 60 to 80 cells per sample (n = 3per group). *p < 0.05, **p < 0.01, ***p < 0.001

**Fig. 11:**
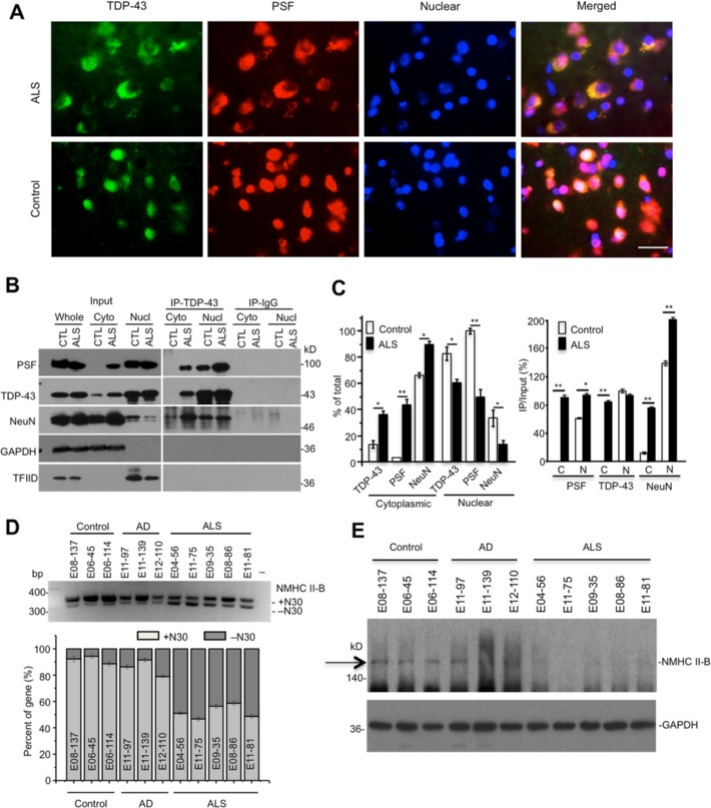
Interactions of TDP-43 with PSF and reduced NMCH II-B in ALS patient brains. **a** Representative images of cytoplasmic mislocalization of PSF and TDP-43 in the ALS patient brain. Double immunofluorescent staining of the brain cortex from ALS patient and non-ALS individual with antibodies to TDP-43 and PSF. Note that ALS patient brain contains cytoplasmic TDP-43 and PSF. Scale bar: 10 μm. **b** Immunoprecipitation of TDP-43 in the cortex tissue of ALS patient showing the presence of TDP-43, PSF, and NeuN in the cytoplasmic fraction. CTL is the sample from a normal individual. Rabbit IgG served as a control immunoprecipitation. Whole: cortex total lysates; Cyto: cytoplasm of cortex lysates; Nucl: nuclear of cortex lysates; **c** The ratios (mean ± SE, n = 3) of immunoprecipitate (IP) to input and cytoplasmic or nuclear proteins to total lysates on western blots are presented. *p < 0.05; **p < 0.01 compared with CTL. **d** RT-PCR gel result (the upper panel) and quantification of the relative levels (the lower panel) of +N30 and -N30 isoforms in the brain cortex samples from 3 normal individuals (control), 3 Alzheimer’s disease (AD), and 5 ALS patients. **e** NMHC II-B western blot analysis of the brain cortex from normal individuals, Alzheimer’s disease (AD) and 5 ALS patients showing decreased expression of NMCH II-B in ALS patient brains

We have obtained ALS patient brain tissues that were found to contain TDP-43 aggregates. Because ALS patient brains had undergone extensive neurodegeneration, we had to examine the neurons that were not degenerated and remained clear cell body morphology in order to assess protein subcellular distribution. We found that those neurons in the ALS brains show more cytoplasmic distribution of PSF and NeuN in the cerebellar cortex and spinal cord than those in the control brains (Fig. 10a). The cytoplasmic colocalization of NeuN and PSF was not seen in the control human brain section and requires the presence of both primary antibodies to NeuN and PSF (Fig. 10b), indicating the specific colocalization in ALS patient brains. Counting the percentage of cells containing cytoplasmic staining of NeuN and PSF also verified that ALS patient brains displayed more cytoplasmic NeuN and PSF (Fig. 10c). Because the postmortem human brain tissues were not well-preserved, there was difficulty in using immuofluorescent staining to analyze these tissues. Despite this, we were also able to find that the ALS patient brain cortex contained more cytoplasmic TDP-43 and PSF than the control individual that was not affected by ALS (Fig. 11a). Immunoprecipitation of TDP-43 from the cytosolic and nuclear fractions of ALS patient and control individual brain cortex tissues revealed that more PSF and NeuN were precipitated by the higher level of cytosolic TDP-43 in the ALS patient sample than the control sample (Fig. 11b and c). All these results from ALS patient samples support the findings from transgenic TDP-43 pig brains for the cytoplasmic distribution of PSF and its interaction with mutant TDP-43.

Next, we wanted to see if NMHC II-B level is also decreased in ALS patient brains. Using RT-PCR to examine alternative splicing of NMHC II-B, we found that tissues from 5 ALS patient cortexes contained more –N30 isoforms than Alzheimer’s disease (AD) and control normal individual brains (Fig. 11d). Western blot analysis of the cerebellar cortex tissues from five ALS patients and their controls also validated the decreased levels of NMHC II-B in the ALS patient brains as compared with the control normal individual brains (Fig. 11e). Some AD patient samples show smear by anti-NMHC II-B staining, perhaps because of tissue degeneration in AD brains. However, the increased –N30 band in ALS patient samples (Fig. 11c) and the short half-life of –N30 (Fig. 9) support the finding that NMHC II-B is decreased in ALS patient brains.

**Fig. 12:**
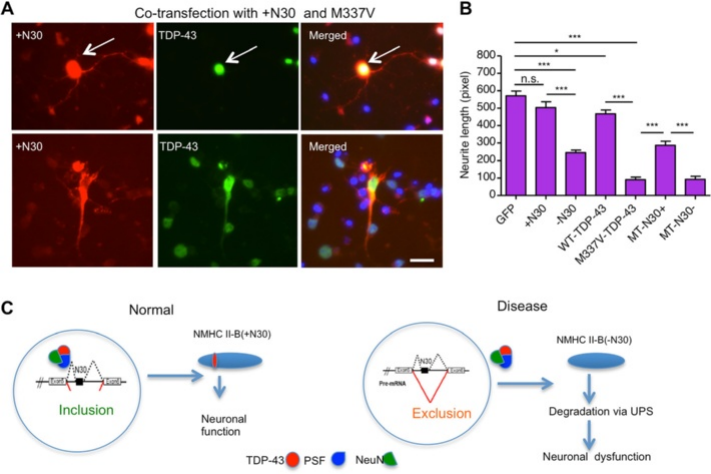
Overexpression of NMHC II-B reduces TDP-43-mediated neurite toxicity. **a** Representative immunofluorescent images of TDP-43 (green), NMHC II-B (red), and nuclei (blue) in primary neurons from the mouse cortex, which were transfected with mutant TDP-43 (M337V) and NMHC II-B (+N30) or NMHC II-B (−N30). Note that transfected TDP-43 produced much intense immunostaining signals over the background level in non-transfected cells. Scale bars: 10 μm. **b** Quantitative data of neurite length of transfected cells. NMHC II-B (+N30) transfection could promote neurite outgrowth and also partly rescue the neurite outgrowth defect caused by mutant TDP-43. The data were obtained by counting more than 300 transfected cells per group in 3 independent experiments. *p < 0.05, **p < 0.01 and ***p < 0.001 compared with TDP-43 transfection alone. GFP: GFP protein; +N30: NMHC II-B (+N30); −N30: NMHC II-B (−N30); WT-TDP-43: wild type TDP-43; MT-TDP-43: mutant TDP-43 (M337V); MT-N30+: mutant TDP-43 (M337V) and NMHC II-B (+N30); MT-N30-: mutant TDP-43 (M337V) and NMHC II-B (−N30). **c** Proposed model of TDP-43 neuronal toxicity. Because of the interaction of TDP-43 with PSF, the cytoplasmic mislocalization of TDP-43 leads to the cytoplasmic distribution of PSF and NeuN and reduces the nuclear function of PSF and NeuN on the splicing of NMHC II-B, leading to the exclusion of N30, which promotes the degradation of NMHC II-B and affects neuronal function

Since NMHC II-B is important for neuronal intracellular trafficking of receptors like NMDA receptor [38–40] and since the lack of NMHC II-B can affect early development [41] and neurite outgrowth [42], we assumed that overexpression of NMHC II-B should diminish TDP-43-mediated toxicity if TDP-43 reduces NMHC II-B in neuronal cells. To test this idea, we transfected mutant TDP-43 with NMHC II-B (+N30) in cultured primary cortical neurons from mice. Overexpression of mutant TDP-43 could significantly reduce neurite growth of cultured mouse cortical neurons, which is largely dependent on intracellular trafficking of a variety of molecules and receptors. This reduced neurite growth could be alleviated by co-transfection with +N30, but not –N30, NMHC II-B (Fig. 12a). Consistently, by measuring the length of neurites of those mouse cortical neurons expressing M337V with +N30 or –N30 NMHC II-B, we found that +N30 NMHC II-B mitigated TDP-43 toxicity on the neuritis outgrowth (Fig. 12b). However, this protection is partial, indicating that TDP-43 mediates toxicity via multiple pathological pathways. Nevertheless, the protection seen with overexpressed NMHC II-B supports the idea that TDP-43 does affect the expression of NMHC II-B.

Based on the finding that mutant TDP-43 interacts with PSF, we proposed a model for the effects of cytoplasmic mislocalization of mutant TDP-43. In this model, pathogenic TDP-43 is distributed in the cytoplasm. Due to its association with PSF and NeuN, the cytoplasmic mislocalization of TDP-43 may lead to the increased levels of PSF and NeuN in the cytoplasm, thereby reducing the nuclear levels of PSF and NeuN. The reduced PSF/NeuN in the nucleus can impair the nuclear function of these proteins and lead to abnormal RNA splicing, such as greater production of –N30 isoform, which is unstable or becomes toxic. The reduced level of NMHC II-B therefore contributes to the neuropathology caused by mutant TDP-43 (Fig. 12c).

## Discussion

TDP-43 contains a bipartite nuclear localization signal sequence and a nuclear export signal that allows nuclear-cytoplasm shuttling [28]. TDP-43 is normally distributed in the neuronal nucleus but pathogenic TDP-43 mislocalizes in the cytoplasm and forms ubiquitinated inclusions in the brains of ALS and FTLD patients [12–14]. The early death, body weight loss, and motor deficit indicate that transgenic mutant TDP-43 affects a variety of brain and body functions, which is consistent with the severe toxicity of mutant TDP-43 in different animals’ models. It has been well documented that the levels of TDP-43 are critical for neuropathology, as loss or overexpression of wild type TDP-43 can cause neurotoxicity and phenotypes in animal models [43, 44]. Although western blot analysis of the homogenized pig brain tissues showed that transgenic TDP-43 is not overexpressed as compared to the endogenous pig TDP-43, it remains to be determined whether transgenic TDP-43 is overexpressed in specific types of neurons. In addition, since our study did not include transgenic pigs that express transgenic wild type human TDP-43, we cannot conclude that the phenotypes in our transgenic pigs are definitively caused by the mutation in TDP-43, the expression levels of transgenic TDP-43, or both.

Since transgenic TDP-43 is expressed ubiquitously under the control of the ubiquitin promoter, it is also possible that the toxicity of mutant TDP-43 in peripheral tissues contributes to the severe phenotypes of TDP-43 pigs, which will require further experiments to investigate. An interesting finding in our study is that transgenic mutant TDP-43 is also distributed in the cytoplasm, a phenomenon that is seen in patient brains. Viral vector expression of TDP-43 in cynomolgus monkey spinal cord was found to result in the cytoplasmic distribution of TDP-43 [45]. Thus, large animals that are much closer to humans may share species-specific regulatory mechanisms that underlie the mislocalization of TDP-43 in the neuronal cytoplasm. However, the expression levels of transgenic TDP-43 could also influence its subcellular distribution, which needs to be considered when interpreting this cytoplasmic distribution phenomenon. Although the mechanisms underlying the cytoplasmic distribution of TDP-43 in pathogenic conditions remain to be investigated, this cytoplasmic distribution is likely to occur in the brain with age or under pathological conditions and is certainly associated with genetic mutations in the TDP-43 gene [46–48]. The pathogenic form of TDP-43, which may be phosphorylated or carry specific conformation that can confer interactions with other proteins, is able to mislocalize in the cytoplasm and forms inclusions in the aged brain. Although mutations in TDP-43 could promote the formation of the pathogenic TDP-43, it is possible that protein interactions with pathogenic TDP-43 in neurons may be different or modulated differently in small and large animals, which can explain the cytoplasmic distribution of TDP-43 in transgenic pig brains. Thus, species-related differences underscore the importance of using large animal models to uncover some important pathological changes that may not occur in small animals.

How cytoplasmic distribution of TDP-43 contributes to neuropathology remains unknown. The cytoplasmic mislocalization of TDP-43 is thought to result in the loss of the nuclear TDP-43 and its sequestration of other proteins in the cytoplasm, leading to theories of loss and gain of function caused by TDP-43 [43, 44]. For example, cytoplasmic mutant TDP-43 was found to impair axonal transport of mRNA granules [6]. Using transgenic TDP-43 pigs, we found the evidence that the cytoplasmic mutant TDP-43 can also cause a mislocalization of RNA splicing factors in the cytoplasm, offering a new explanation for the role of cytoplasmic pathogenic TDP-43.

Studies of gene expression in TDP-43 transgenic animal models indicate that mutant TDP-43 affects the splicing and expression of a large number of genes [5, 7, 15, 49]. However, it is likely the effect of mutant TDP-43 on gene expression may not be the same in different cell types and different disease stages. A new finding from our study is that TDP-43 can interact with the splicing factor PSF. The binding region was found in the C-terminal region of TDP-43, consistent with the fact that this C-terminal region associates with many other heterogeneous nuclear ribonucleoproteins (hnRNP) family members. The association of mutations in this C-terminal region with ALS and FTLD [9, 10] also supports the idea that the abnormal interactions of TDP-43 with other hnRNPs or proteins can affect RNA splicing to induce neuropathology. Also, mutations in TDP-43 can alter its subcellular distribution and stability [8, 50, 51], leading to the nuclear and cytoplasmic effects that account for many kinds of abnormal gene expression and RNA splicing.

In TDP-43 transgenic pig brains, we saw diffuse staining of TDP-43 in the cytoplasm without clear aggregates. The formation of cytoplasmic aggregates may be age-dependent or may only occur in old pig brains. The lack of obvious cytoplasmic TDP-43 aggregates supports the idea that soluble TDP-43 is toxic in our TDP-43 transgenic pigs. In fact, this idea has been suggested by the earlier finding that deleting the NLS in mutant TDP-43 could cause the cytoplasmic distribution of mutant TDP-43 and severe phenotypes in transgenic mice without aggregates [52]. Also, aberrant RNA splicing was recently found to occur without aggregation and loss of nuclear TDP-43 [53]. This idea is also supported by the interactions of soluble TDP-43 with PSF/NeuN and abnormal splicing of MNHC II-B in transgenic pig and ALS patient brains. The expression of MNHC II-B is critical for neuronal development [38–40]. Decreased levels of MNHC II-B and its point mutations are found to cause neuronal toxicity and phenotypes in mice and humans [53, 54], making MNHC II-B a good candidate for investigating how TDP-43 mediates neuronal function via affecting NMHC II-B gene slicing and expression.

According to our proposed model, the mislocalization of TDP-43 in the cytoplasm and its interactions with PSF/NeuN may contribute to the cytoplasmic distribution of PSF and NeuN, thereby reducing their effect on RNA splicing in the nucleus. It is possible that the interaction of mutant TDP-43 with PSF/NeuN in the nucleus can impact the function of PSF/NeuN, as the reduced neurite outgrowth of transfected cells showing both cytoplasmic and nuclear distribution of mutant TDP-43 can be alleviated by NMHC II-B. However, TDP-43 is known to interact with a large number of proteins. The contributions of the interactions of TDP-43 with its partners to ALS pathology may depend on cell types, subcellular regions, and species-related factors, which could confer differential posttranslational modifications to modify these interactions.

## Conclusions

Using nuclear transfer technology, we established transgenic pigs expressing mutant TDP-43. Like other transgenic TDP-43 animal models, transgenic TDP-43 pigs show severe neurological phenotypes, supporting a toxic gain of function of mutant TDP-43 in large mammalian animals. We also found that mutant TDP-43 is distributed in the cytoplasm in the transgenic pig brain, a phenomenon that is seen in patient brains. By focusing on the TDP-43-PSF interaction and its related RNA splicing function, we provide new evidence for species-dependent cytoplasmic distribution of mutant TDP-43 and a new insight for how the cytoplasmic accumulation of mutant TDP-43 can contribute to abnormal gene expression. These findings should also have implications for identifying other pathological pathways and interacting proteins involved in ALS and FTLD and help uncover therapeutic targets, as well.

## Methods

### Plasmids and antibodies

pBudCE4.1-PSF was obtained from Dr. V.M. Kalscheuer’s lab [55]. pEGFP-NMHC II-B (−N30) was obtained from Addgene, and pEGFP-NMHC II-B (+N30) was constructed by inserting +N30 sequences into pEGFP-NMHC II-B (−N30) using primers and PCR. The shRNA of NMHC II-B (TL711338A, TL711338B, TL711338C and TL711338D) were obtained from Origene. Other constructs of GST-TDP-43(1-414-M337V), GST-TDP-43(1–251), GST-TDP-43(264–414), GST-TDP-43(264-414-M337V), His-PSF(1–707), His-PSF(297–369), His-PSF(444–495), His-PSF (496–707), His-PSF(444–707), pRK-TDP-43(M337V), pRK-C-TDP-43, pRK-C-TDP-43(M337V), pEGFP-TDP-43(M337V) and pEGFP-NMHC II-B (+N30) were generated using the above plasmids or using PCR primers to amplify the first strand cDNAs of human brain tissues and constructed in the pRK5 or pCDNA3 vector. For antibodies, rabbit polyclonal antibody to TDP-43 was raised against GST-TDP-43 at the Institute of Genetics and Developmental Biology in Beijing and was used for immunoprecipitation. Antibodies to PSF (Ab38148), P84 (5E10), Flag (Ab21536) and NMHC II-B [3H2] (ab684) were obtained from Abcam. Other antibodies purchased include: NeuN (mAb377) from Millipore; TDP-43 (12892-1-AP) from Proteintech; TDP-43 (G400) from Cell Signaling; TDP-43 (M01) from Abnova; GFP (GT859) and FUS (GTX63610) from GeneTex; GAPDH (G-9), beta-actin (C-4), Adpatin B (N-19), hnRNPK (D-6), TFIID (N-12), GST (A-6) and His (H-15) from Santa Cruz Biotechnology.

### Transgenic pigs

Animal use followed the NIH Guide for the Care and Use of Laboratory Animals. The animal use protocol was approved by the Institutional Animal Care and Use Committees (IACUC) at Guangzhou Institute of Biomedicine and Health (GIBH), Chinese Academy of Sciences. TDP-43 (M337V) cDNA [56] was tagged with the flag epitope at its N-terminal region and fused with EGFP via F2A, a peptide sequence that is self-cleaved to separate TDP-43 from EGFP in cells. EGFP served as a reporter for selecting TDP-43 transfected cells. The cDNA was inserted into the vector (pCAG-Neo) containing the neo-resistant gene and the CAG promoter were replaced with the human ubiquitin (hUPC) promoter. The constructed TDP-43 vector (phUBC-TDP-43(M337V)-F2A-GFP) was then transfected via electroporation (Gene PulserXcell, Bio-Rad, Hercules, CA, USA) into primary pig fetal fibroblasts isolated from a 35-day fetus of a female Tibet miniature pig. The fetal fibroblasts were cultured in Dulbecco’s modified Eagle’s medium (DMEM, HyClone, Logan, UT, USA) supplemented with 15 % fetal bovine serum (FBS, HyClone, Logan, UT, USA) and 1 % (v:v) penicillin/streptomycin (10,000 U/ml penicillin, 10,000 μg/ml streptomycin; GIBCO-BRL, Grand Island, NY, USA) at 39 °C in an incubator with 5 % CO_2_. After 48 h, 500 μg/ml G418 (Merck, Darmstadt, Germany) was added to the medium to select transfected cell colonies, and the plates were incubated in media containing G418 for approximately 10 d. The surviving cell colonies were selected and propagated in a fresh 48-well plate. Colonies that proliferated well were then expanded and screened for the expression of TDP-43.

The ovaries of pigs were collected from long white pigs at a local slaughterhouse. The cumulus-oocyte complexes were isolated and cultured as described previously [26, 27]. After 42–44 h of culture, oocytes were separated from cumulus cells by vigorous vortex for 4 min in TL-Hepes supplemented with 0.1 % polyvinyl alcohol (P8136; Sigma) and 0.1 % hyaluronidase (H3506; Sigma). Denuded mature oocytes were enucleated and injected with a single donor cell into the perivitelline space of the oocyte in contact with the oocyte membrane. Fusion and activation were induced simultaneously with 2 successive DC pulses of 1.1 kV/cm for 30 ms using an Electro Cell Manipulator 200 (Genetronics, San Diego, CA, USA). Reconstructed embryos (50–100) were transferred to 500 μl of culture medium covered with mineral oil in a four-well multi dish and cultured in 5 % CO_2_ for 18–22 h at 39 °C. The embryos were then surgically transferred into the oviduct of a surrogate female on the first day of standing estrus. Pregnancy status was monitored using an ultrasound scanner between days 30–35 post-transplantation. Transgenic piglets were identified by PCR using primers (forward primer: 5’-TTGAACTATGCGCTCGGGGTTG-3’ and reverse primer: 5’-GTTCGGTTGTTTTCCATGGGAGAC-3’) and western blot analysis with anti-TDP-43 antibodies.

### Animal behavioral studies

The movement capabilities of transgenic pigs were tested by treadmill running as described in our recent study [27]. A suitable cage was placed onto the treadmill to keep the pig and make it run on the conveyer belt. Pigs were trained 3 times for running on treadmill and then were tested from the age of 3 months. The speed of treadmill was kept at 3.0 km/h for 1 min when the pig ran on it. Non-transgenic littermates or age- and body weight-matched non-transgenic pigs were used as controls. Due to their severe phenotypes, TDP-43 transgenic pigs often died in the course of training or formal test of their motor function such that we did not test all TDP-43 transgenic pigs.

### Human tissue acquisition

Human cortex and spinal cord tissues were obtained and archived via an institutional review board and Health Insurance Portability and Accountability Act-compliant process at neuropathology/histochemistry core of Emory University. Autopsies were performed following death with post-mortem interval of 6–13 h. The spinal cord and cortex tissues were obtained from the postmortem brains of ALS patients (04–56, E11-75, E09-35, E-86, and E-81) who died at 67–74 years of age and were confirmed to have TDP-43 aggregates via post-mortem histologic analysis. Non-ALS tissues were obtained from neurologically unaffected patients (E08-137, E06-45, and E06-114) and Alzheimer’s disease patients (E11-97, E11-139, and E12-110) who died at 53–92 years of age. For immunofluorescence studies, tissues were fixed in formalin and embedded by paraffin, sectioned, and stored at room temperature (16-25 °C). For Co-IP and RT-PCR analysis, tissue segments stored at −80 °C were used.

### GST pulldown assays

GST-TDP-43 recombinant fusion proteins were produced in bacteria (BL21) and purified with glutathione-sepharose beads (Sigma). Pig cortex tissues (0.1 g/ml) in NP-40 lysis buffer (50 mM Tris pH7.4, 50 mM NaCl, 0.1 % Triton X-100, 1 % NP40) were homogenized using a Teflon homogenizer for 30 strokes. The lysates were added with protease inhibitor cocktail (Sigma, P8340) plus proteasome inhibitor and kept on ice for 30 min. Lysates were sonicated for 10 s and centrifuged at 1000 × g for 2 min. The supernatants (5 μg/μl protein) were pre-cleaned by incubating with 100 μg GST protein and 30 μL glutathione-sepharose beads for 4 h at 4 °C and then subjected to GST-TDP-43 pull-down assay. Analysis of the GST-TDP-43 pull down bands was performed by AB SCIEX TripleTOF® 5600 system at Institute of Genetics and Developmental Biology in Beijing.

### Co-immunoprecipitation and western blot

Cultured cells or brain tissues were homogenized in NP-40,1 × PBS or RIPA buffer with 1× protease inhibitor as previously described [57]. For immunoprecipitation, the lysates were centrifuged at 800 × g for 5 min, and supernatants were incubated with antibody and Protein A beads (Sigma-Aldrich) at 4 °C overnight, followed by washing with lysis buffer 4 times. The samples were subjected to 4-12 % SDS-PAGE (Invitrogen) and detected with SuperSignal West Dura Extended Duration Substrate (Thermo). For RNase treatment, pig cortex lysates were incubated with 200 μg/ml RNase A for 30 min at 25 °C before immunoprecipitation.

### Subcellular fractionations

The cytosolic fraction was obtained by centrifuging the brain lysates at a high speed 15000 × g for 15 min, the supernatant was used as cytoplasmic for Western Blot and Co-Immunoprecipitation. For nuclear fractionation, we added 1000 μl of NP-40  buffer to 0.1 g pig brain cortex tissue on ice for 10 min. The tissues were then homogenized with a glass homogenizer for 30 strokes and sonicated of 15 s. The lysates were centrifuged at 4 °C at 800 × g for 10 min. The pellets were re-suspended in 374 μl of buffer (5 mM HEPES, 1.5 mM MgCl2, 0.2 mM EDTA, 0.5 mM DTT, 26 % glycerol (v/v), pH 7.9) with 26 μl of 4.6 M NaCl to give the final concentration at 300 mM NaCl and then homogenized with 20 full strokes in Teflon homogenizer on ice and sonicated for 10 s. The homogenized samples were kept on ice for 20 min and then centrifuged at 24,000 × g for 20 min at 4 °C. The supernatant was used for western blotting or Immunoprecipitation.

### Cell cultures

PC12 cells stably expressing flag-TDP-43-EGFP were described in our recent study [56]. HEK293T and SK-N-SH cells were purchased from the ATCC and cultured according to the guidelines recommended by the ATCC. All cells were maintained at 37 °C with 5 % CO_2_. PC12 cells were maintained in DMEM/F12 medium with 5 % fetal bovine serum and 10 % horse serum, 100 U/ml penicillin at 37 °C in an atmosphere of 5 % CO_2_ and 95 % humidity. NGF was used at 100 ng/ml (N6009, Sigma) to induce neurite extension for the PC12 cells.

Culture of primary neurons from mouse brain cortex was performed as described previously [57]. Briefly, cortical neurons were isolated from the cortex of postnatal day 1 mice. Dissected tissue was treated with 0.0625 mg/ml trypsin and 0.0625 mg/ml DNase (Life Technology) in 1 × HBSS buffer without calcium and magnesium for 10 min at 37 °C, followed by triturating with a 1-ml pipette tip for 20 times. Cells were then washed once with the tissue culture medium and spun down at 1500 g for 3 min. Cells were placed on top of a layer of astrocytes and grown initially in a 50 % glial-conditioned medium [DMEM containing 0.25 % glucose, 2 mM glutamate, 10 % FCS, 500 nm insulin, 1 × vitamin mixture (M6895; Sigma), and 1 % antibiotic–antimycotic (Invitrogen)]. The cells were then cultured in neurobasal/B27 medium.

### Immunofluorescence, immunohistochemistry, and quantification

Sections from human or pig brains were fixed in 4 % paraformaldehyde/1 × PBS, embedded in paraffin or Tissue ® Tek O.C.T compound (SAKURA), and sectioned to 10 or 40 μm slices. The paraffin embedded sections were deparaffinized with xylene and sequential ethanol (70 %, 80 %, 100 % X 2 times) and rehydrated with water. Rehydrated slices were heat-treated for antigen retrieval in citrate buffer. The treated slices were then stained by antibodies to TDP-43, NeuN, and PSF and detected using DAB staining. Images of micrographs were taken using a Zeiss microscope (Axiovert 200 MOT).

To quantify neurons expressing transgenic TDP-43 in transgenic pig brains and ALS patient brains, we used 5–8 images obtained with 20 × N.A. 0.8 objective per section and 5 sections per group (n = 3-5 samples per group). To quantify cultured cells, we examined more than total 300 cells per group in at least three independent experiments. To measure the lengths of neurites of cultured neurons, imaged cells were selected at random, and all visible processes of selected neurons were imaged. The transfected neurons whose neurites could be clearly identified were selected to measure the lengths of their neurites using a fluorescent microscope of ZEISS Axiovrert 200 M microscope. The images were analyzed by measuring the longest branch of each neurite and the relative length was quantified as pixel using NeuronJ plugin image software, which is used for quantification of elongated structures.

For analyzing histological changes in the muscle sections, a minimum of 3 random fields (corresponding to 200–300 cross-sectioned fibers) were photo micrographed for gastrocnemius muscle of non-transgenic and TDP-43 transgenic pigs; all type fibers were analyzed for their size using Scion Image software (v. alpha 4.0.3.2, Scion, Frederick, MD).

### Statistical analysis

For statistical analysis, data were obtained from three or more independent experiments. Statistical analysis was performed using Microsoft Excel and Student’s *t*-test (two tailed) for comparing two different groups. A value of *p* < 0.05 or *p* < 0.01 was indicated with one asterisk (*) or two asterisk (**), which represent statistically significant.

### Ethical approval

Animal use followed the NIH Guide for the Care and Use of Laboratory Animals. The animal use protocol was approved by the Institutional Animal Care and Use Committees (IACUC) at Guangzhou Institute of Biomedicine and Health (GIBH), Chinese Academy of Sciences. Human cortex and spinal cord tissues were obtained and archived via an institutional review board and Health Insurance Portability and Accountability Act-compliant process at neuropathology/histochemistry core of Emory University.

## Abbreviations

TDP-43: TAR DNA-binding protein 43
PSF: Splicing factor, proline- and glutamine-rich
NeuN: Feminizing Locus on X-3, Fox-3, Rbfox3, or Hexaribonucleotide Binding Protein-3
NMHC II-B: nonmuscle myosin heavy chain II-B
SOD1: Superoxide dismutase [Cu-Zn] also known as superoxide dismutase 1
ALS: Amyotrophic lateral sclerosis
FTLD: Frontotemporal lobar degeneration
hnRNP: heterogeneous nuclear ribonucleoproteins
